# Blunt traumatic right coronary artery dissection presenting with second-degree atrioventricular block and late-onset severe cardiogenic shock

**DOI:** 10.1186/s12872-022-02784-6

**Published:** 2022-07-30

**Authors:** Maria Paparoupa, Lenard Conradi, Malte Lennart Warncke, Lennart Well, Christoph Burdelski, Christopher Cramer, Hanno Grahn, Mathias Kubik, Stefan Kluge

**Affiliations:** 1 Department of Intensive Care Medicine, University Medical Center Hamburg-Eppendorf, Martinistr.52, 20246 Hamburg, Germany; 2 Department of Cardiovascular Surgery, University Heart and Vascular Center Hamburg, Martinistr.52, 20246 Hamburg, Germany; 3 Department of Diagnostic and Interventional Radiology and Nuclear Medicine, University Medical Center Hamburg-Eppendorf, Martinistr.52, 20246 Hamburg, Germany; 4 Department of Trauma and Orthopedic Surgery, University Medical Center Hamburg-Eppendorf, Martinistr.52, 20246 Hamburg, Germany; 5 Department of Cardiology, University Heart and Vascular Center Hamburg, Martinistr.52, 20246 Hamburg, Germany

**Keywords:** Blunt chest trauma, Acute myocardial infarction, Right ventricular insufficiency, Computed tomography coronary angiography, Surgical coronary revascularization

## Abstract

**Background:**

Blunt chest injury may induce several cardiovascular traumata, requiring immediate care. Right coronary artery dissection (RCA) is an extremely rare sequela in this setting and is associated with high mortality, if it remains undiagnosed.

**Case presentation**

We present the case of an RCA dissection after blunt chest trauma in a 16-year-old patient, who initially presented with a second-degree atrioventricular block as solitary manifestation on admission. Typical electrocardiographic findings, such as ST segmental changes or pathological Q waves were absent. Serial echocardiograms excluded segmental motion abnormalities, pericardial effusion or right ventricular strain. Nevertheless, a complementary computed tomography coronary angiography revealed this potentially lethal condition several hours later. The patient underwent an emergency surgical myocardial revascularization under the circulatory support of veno-arterial extracorporeal membrane oxygenation and suffered a prolonged right ventricular insufficiency with severe late-onset cardiogenic shock, due to an extensive myocardial infarction of the inferoseptal ventricular wall.

**Conclusion:**

Right coronary artery dissection after high-speed blunt chest injury constitutes a diagnostic challenge, especially in the absence of typical electrocardiographic and echocardiographic findings in young patients. This condition may dramatically deteriorate in time, leading to severe cardiogenic shock and life-threatening arrhythmias.

## Background

Blunt chest injury is usually caused by high-speed motor vehicle accident, falling from height, blunt instrument injury or physical assault [1]. The true incidence of this entity is unknown, ranging between 8 and 71% in different literature reports [2]. As a result of blunt chest trauma many life-threatening conditions may occur, requiring immediate care. Chest wall and pulmonary injuries range from rib fractures and frail chest to pneumothorax, hemothorax, pulmonary contusion and tracheobronchial laceration [1]. Blunt cardiac injury may also encompass several traumata, such as valvular rupture, myocardial contusion, aneurysm or rupture and coronary artery dissection, thrombosis or rupture. The clinical manifestations vary from asymptomatic and transient arrhythmias, to acute myocardial infarction (AMI) and sudden death [3].

Blunt trauma-induced coronary artery (CA) dissection is an extremely rare sequela, associated with increased mortality, requiring a high clinical awareness and extensive diagnostic work-up. Traumatic coronary dissection may result in intraluminal thrombosis and coronary occlusion with subsequent acute coronary syndrome and life-threatening arrhythmias [4].

The diagnosis of CA dissection in the setting of chest trauma is challenging. While conventionally coronary angiography has been the diagnostic tool of choice [4, 5], modern imaging techniques such as computed tomography coronary angiography (CTCA) can further improve diagnostic accuracy and help optimize the treatment strategy [6]. This fast and non-invasive diagnostic method provides good quantitative and qualitative assessment of the coronary arteries and the aortic root. CTCA can accurately identify the location and extent of coronary injury and differentiate between plaque rupture, thrombotic material, dissection or external compression [3].

The ideal treatment modality for managing CA dissection has not been established yet, with case reports revealing a variety of treatment strategies [6]. Possible options include invasive techniques (percutaneous transluminal coronary angioplasty), surgical revascularization, thrombolysis (at the risk of deterioration of the dissection) and conservative treatment. The conservative approach was reported to be appropriate for cardiopulmonary stable patients, with resolution of symptoms and limited infarction [7].

We present a case of right coronary artery (RCA) dissection after blunt chest trauma in a 16-year-old patient and we would like to stress the importance of having a high index of suspicion for this condition in the setting of blunt chest injury. Early recognition and treatment are crucial for the survival in patients suffering from this pathology.

## Case presentation

A 16-year-old patient was admitted to the emergency department of our medical center to treat injuries incurred as a result of a traffic accident. He suffered a left thoracoabdominal collision, while driving a scooter, with a passing vehicle at high speed. Initial evaluation raised the clinical suspicion of a left sided tension pneumothorax, with subsequent blind insertion of two thoracic tubes on his way to the hospital.

On admission, the patient was already intubated and mechanically ventilated due to respiratory insufficiency. Further examination revealed numerous abrasions of the chest and abdomen and superior vena cava syndrome (SVCS), likely secondary to the incomplete resolution of tension pneumothorax. A polytrauma computed tomography (CT) scan was performed and revealed multiple rib fractures, pulmonary contusion, the undrained left sided hemopneumothorax and a splenic laceration Grade I. The patient underwent an urgent left-side thoracotomy, with drainage and intraoperative reposition of the thoracic tubes, as well as explorative laparotomy, where active bleeding into the abdominal cavity and hollow-organ perforation were excluded. Splenectomy was not necessary and the patient was transferred to the intensive care unit (ICU) for postoperative cardiopulmonary monitoring.

**Fig. 1:**
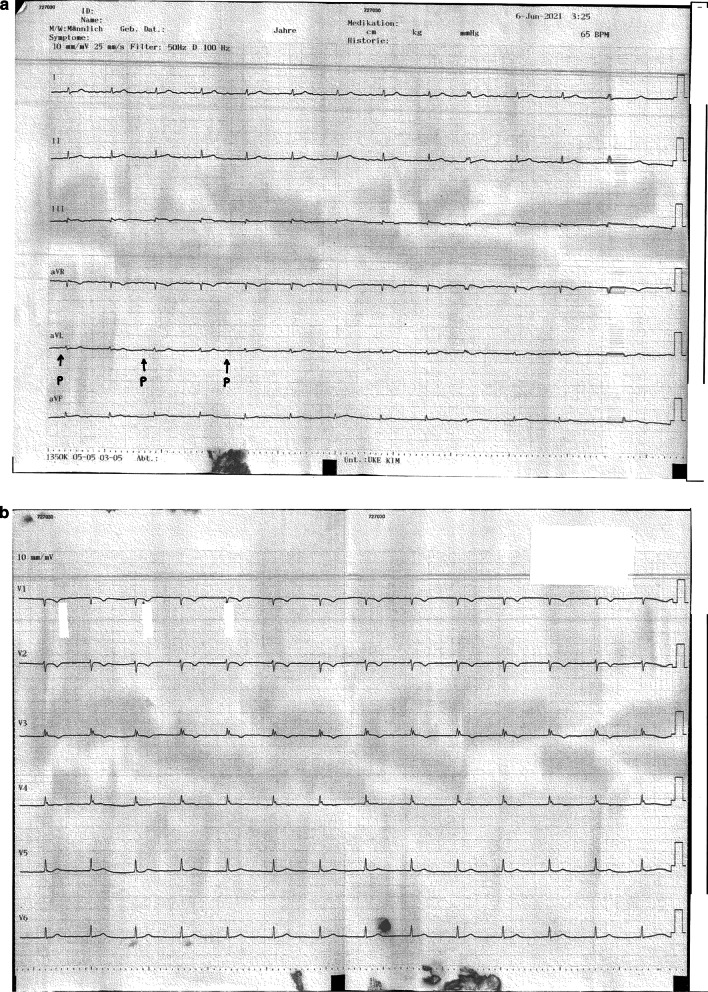
**a**, **b** Electrocardiogram with a second-degree atrioventricular block and low-voltage QRS complexes in all leads

The patient’s circulatory and respiratory function remained impaired so that mechanical ventilation and infusion of vasopressors (norepinephrine) had to be continued. His heart rate was normal (circa 70 beats per minute), although a second-degree atrioventricular block (AVB) was present. A 12-lead electrocardiogram (ECG) confirmed the presence of a second-degree AVB type Mobitz I (Weckenbach) and revealed low-voltage QRS complexes in all leads, without ST segment abnormalities or pathological Q waves (Fig. 1a, b).

The patient suffered a severe trauma with hypovolemia and aseptic inflammatory syndrome, which required a high cardiac output to be compensated. The heart rate of circa 70 beats per minute, due to the AV-block, was not high enough to achieve that. We initiated a temporary right ventricular pacing, in order to elevate the heart rate of the patient and subsequently his cardiac output. Drugs or dyselectrolytemia were excluded as a causative reason of the block. Cardiology was consulted because of elevated high-sensitivity cardiac troponin I (hs-cTnI) (19,706 pg/ml, 99th percentile males 53.5 pg/ml, Siemens Atellica® IM Analyzer).

The bedside echocardiography demonstrated a normal right and left ventricular wall motion and excluded segmental motion abnormalities, pericardial effusion or right ventricular strain. Based on these findings, myocardial contusion was suggested as the most likely diagnosis and, subsequently, decision against coronary angiography was made. Severe cardiac contusion is characterized by a non-ischemic post-traumatic irregular myocyte necrosis associated with epicardial haemorrhage extending in pyramidal fashion intramurally or even transmurally. The right ventricle (RV) is most commonly injured because of its position behind the sternum [8].

**Fig. 2:**
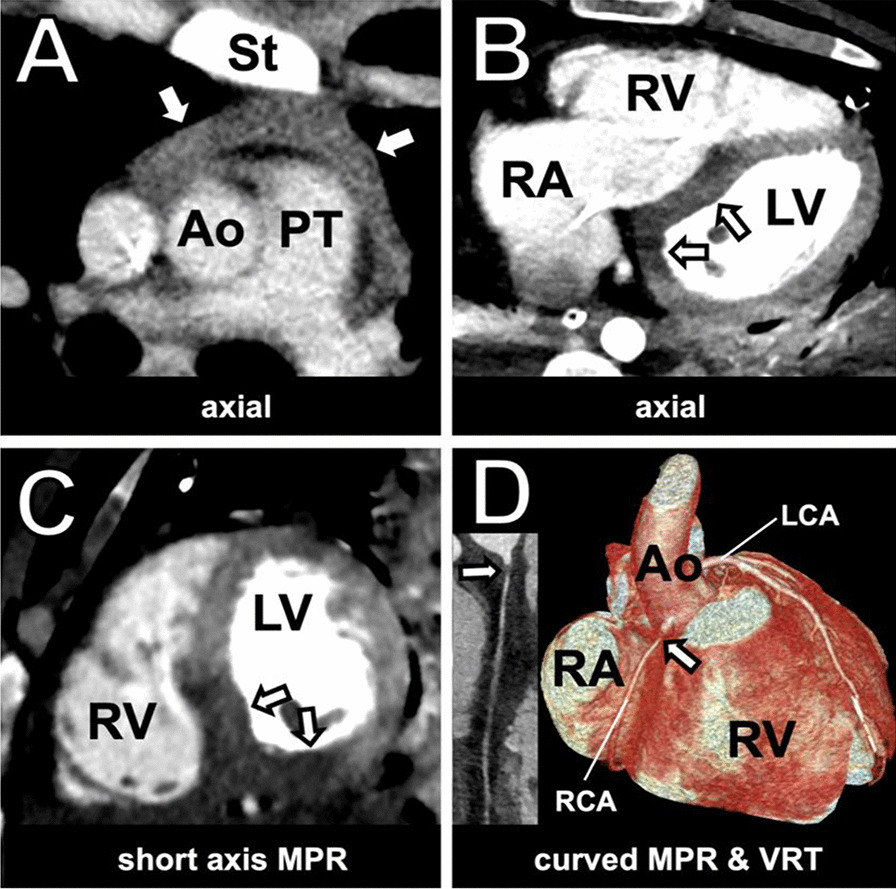
Contrast-enhanced CT and CTCA showing pericardial effusion, right heart strain and proximal RCA dissection. **A** Contrast-enhanced CT showed a progressive pericardial effusion (white arrows). **B**, **C** Massive right heart strain and inferoseptal myocardial infarction (white arrows). **D** Coronary CT angiography revealed a short proximal discontinuation (white arrows) of the RCA, highly suggestive of dissection

Three hours later the patient developed a self-limiting ventricular tachycardia and, despite complete absorption of the tension pneumothorax, clinical deterioration of the underlying SVCS could be observed. An emergency whole body CT scan and CTCA confirmed the diagnosis of an underlying RCA dissection with the concurrent presence of a 2 cm pericardial effusion, positioned ventrally to the aortic root, a region of infarction in the inferoseptal ventricular wall and massive right ventricular strain with tricuspid regurgitation (Fig. 2). Pulmonary artery embolization and Stanford type A aortic dissection were excluded. The tension pneumothorax was completely resolved and the tip of the thoracic tubes was located apically in the left pleura.

The patient underwent an emergency beating-heart surgical myocardial revascularization under the circulatory support of veno-arterial extracorporeal membrane oxygenation (VA-ECMO) in severe cardiogenic shock. The intraoperative findings were a non-hemorrhagic pericardial effusion and a large hematoma of the right ventricular wall. Right ventricular function eventually recovered after 3 weeks and the VA-ECMO was explanted, despite initial failing weaning attempts. The patient could be mobilized on floor level and he was completely weaned from mechanical ventilation and renal replacement therapy. At 6-week follow-up, echocardiography showed improved right ventricular function and invasive hemodynamics revealed normalized right-and left sided filling pressures, despite ongoing cardiac hypoperfusion syndrome (CI 1.9 (L/min)/m^2^). The clinical status of the patient was considered to be stable and he was sent to rehab with closed follow-up examination in our outpatient clinic.

## Discussion and conclusions

RCA dissection is a rare clinical entity and may occur spontaneously or secondary to Stanford type A aortic dissection or large calcified aortic valve mass inserting into the right coronary artery [9–11]. The most relevant traumatic etiologies include diagnostic and therapeutic coronary catheterization and blunt chest trauma after high-speed motor vehicle collision [4, 12]. Extremely rare is the dissection after delivery or low impact chest trauma during sport activities [13, 14]. Apart from the causal mechanism, diagnostic work-up of this condition remains challenging and early diagnosis and treatment is crucial for the patient’s outcome.

The main clinical presentation of RCA dissection is AMI, as the dissection membrane or thrombotic material may lead to subtotal or total occlusion of the arterial lumen [4, 5]. Angina symptoms may be absent in patients with severe traumatic injuries, who need extensive cardiopulmonary support under general anesthesia [10, 13]. The asymptomatic clinical course consists a major risk factor for delayed diagnosis in this population.

Typical electrocardiographic findings by RCA dissection are ST segmental abnormalities and life-threatening arrhythmias [5, 13, 15]. Complete AVB, due to the obstruction of blood supply to the atrioventricular conduction system, has been already described in the literature [5, 10]. To our knowledge, this is the first report of a second-degree atrioventricular block in a patient with traumatic RCA dissection after high-speed blunt chest trauma. Another case of a 2:1 atrioventricular block as a result of RCA dissection during a coronary angiography has been reported before [16]. The anatomic relevance between atrioventricular node artery, shown to be originated from the right coronary artery in 73% of the examined cases, explains this correlation [17].

Despite the already ongoing extensive myocardial hypoperfusion, ST segmental changes or pathological Q waves, were absent in our case. Bedside echocardiography excluded right ventricular strain and segmental motion abnormalities of the left and right ventricular wall. Because typical electrocardiographic and echocardiographic signs of a myocardial infarction were absent, elevated hs-cTnI was interpreted as manifestation of a myocardial contusion. Although low-voltage QRS complexes were present in all leads, pericardial effusion could be definitely excluded in serial echocardiographic controls. A similar case of sparse findings by RCA dissection has been described in a basketball player, after a low-energy blunt chest trauma. He presented with an underlying sinus bradycardia, first-degree AVB and occasional isorhythmic atrioventricular dissociation, but no ischemic ST changes [18].

However, we decided for a complementary CT scan of the chest and abdomen three hours later, as clinical deterioration of the SVCS and a self-limiting ventricular tachycardia occurred. A coronary pathology was not in our differential diagnosis at that point. We rather suspected an undiagnosed mediastinal pathology, like a mediastinal hematoma or pneumomediastinum, causing an upper inflow congestion. The contrast-enhanced CT scan revealed a new pericardial effusion, massive right heart strain and inferoseptal myocardial infarction and a complementary CTCA confirmed the underlying RCA dissection. We believe, in retrospect, that the absence of echocardiographic and electrocardiographic findings, by admission and several hours later, was due to prolonged myocardial compensation by a young patient. Though, physiological compensation mechanisms of shock, like sinus tachycardia, were absent in our case, probably due to the underlying second-degree AVB.

The patient underwent an emergency beating-heart surgical myocardial revascularization under the circulatory support of VA-ECMO. The implementation of extracorporeal membrane oxygenation in patients with acute heart failure as a result of a right coronary dissection has been already described in the literature [19]. A post-surgical coronary angiography demonstrated a successful blood supply of the venous graft and the other coronary arteries. Still, the extensive myocardial infarction caused a prolonged right ventricular insufficiency.

Dissection of the RCA after high-speed blunt chest injury constitutes a diagnostic challenge, especially in cases of sparse electrocardiographic and echocardiographic findings in young patients and may lead to dramatic deterioration in time, severe prolonged cardiogenic shock and life-threatening arrhythmias.

## Data Availability

All data generated or analysed during this study are included in this published article (and its supplementary information files).

## Abbreviations

AMI: Acute myocardial infarction
AVB: Atrioventricular block
CA: Coronary artery
CI: Cardiac index
CTCA: Computed tomography coronary angiography
CT scan: Computed tomography scan
ECG: Electrocardiogram
hs-cTnI: High-sensitivity cardiac troponin I
ICU: Intensive care unit
RCA: Right coronary artery
SVCS: Superior vena cava syndrome
VA-ECMO: Veno-arterial extracorporeal membrane oxygenation
